# The Topography of Poverty in the United States: A Spatial Analysis Using County-Level Data From the Community Health Status Indicators Project

**Published:** 2007-09-15

**Authors:** James B Holt

**Affiliations:** Division of Adult and Community Health, Centers for Disease Control and Prevention

## Abstract

Socioeconomic and health-related data at the county level are now available through the Community Health Status Indicators (CHSI) database. These data are useful for assessing the health of communities and regions. Users of the CHSI data can access online reports and an online mapping application for visualizing patterns in various community-related measures. It also is possible to download these data to conduct local analyses. This paper describes a spatial analysis of poverty in the United States at the county level for 2000. Spatial statistical techniques in a geographic information system were used to quantify significant spatial patterns, such as concentrated poverty rates and spatial outliers. The analysis revealed significant and stark patterns of poverty. A distinctive north–south demarcation of low versus high poverty concentrations was found, along with isolated pockets of high and low poverty within areas in which the predominant poverty rates were opposite. This pattern can be described as following a *continental poverty divide*. These insights can be useful in explicating the underlying processes involved in forming such spatial patterns that result in concentrated wealth and poverty. The spatial analytic techniques are broadly applicable to socioeconomic and health-related data and can provide important information about the spatial structure of datasets, which is important for choosing appropriate analysis methods.

## Introduction

The release of the Community Health Status Indicators (CHSI) database provides ready access to a rich compilation of data for researchers and individuals interested in the health of communities (1). CHSI data cover a wide range of county-level attributes that describe the sociodemographic context in which people live. These attributes, often referred to as *social determinants of health* (2), have been found to have important proximate and distal influences on health-risk behaviors and health outcomes for individuals. With CHSI, many individuals for the first time will have convenient one-stop access to these data. Heitgerd et al have developed an Internet mapping application, powered by a geographic information system (GIS), which will provide a means to explore the CHSI data through geospatial visualization (3). This innovation will provide users with ready-made tools to map their data in comparison with "peer" counties as well as neighboring counties. This added mapping application introduces a spatial component that is not otherwise available.

Many CHSI data users will likely want to explore more fully the spatial structures of the data. They may be interested in a particular indicator of socioeconomic status (SES) and whether their own county's performance on this measure is better or worse than the performance of neighboring areas. They may wish to know whether they are part of a larger spatial concentration of similar conditions or whether they represent a spatial outlier. Knowing the answers to these questions may help researchers and policymakers to devise more in-depth research questions when planning effective intervention strategies. Although spatial analysis can be attempted visually in rudimentary form using an Internet-based mapping application, specialized GIS *and* spatial statistics software are needed to fully leverage the spatial component of the data. This paper describes one basic example of how users can explore the spatial structure of one SES variable (poverty) and make some informed statements about the spatial patterns and concentrations of the variable. In a sense, this type of analysis is quite similar to descriptive epidemiology, but with the addition of a spatial component.

I have chosen to illustrate poverty because its influence on health is significant, unequivocal, and well-documented. Recent research examples include Brimblecombe et al (4), Braveman and Tarimo (5), Krieger et al (6), Kobetz et al (7), Gold et al (8), and Krieger et al (9). Individuals living in poverty tend to be exposed to social, psychosocial, and physical factors associated with increased morbidity and mortality more than do middle-class or wealthy people. These factors include acute and chronic stress, overburdened or disrupted social supports, material deprivations, and exposure to hazards such as toxins or pollutants in the physical environment. The psychosocial stresses often lead to increases in unhealthy behaviors and a lowered ability to access health information, health services, or technologies that could protect them from exposure to health hazards or reduce their risk from such exposure. The negative influences resulting from poverty are often exacerbated for people from racial and ethnic minorities, such as African Americans, Hispanics, and American Indians, because their poverty often extends throughout their entire lifespan, thus suggesting a cumulative adverse health effect from being persistently disadvantaged (10).

## Methods

Poverty data were downloaded from the CHSI database in dBase (dataBased Intelligence Inc, Vestal, New York) format and imported into ArcGIS 9.2 (Environmental Systems Research Institute, Redlands, California), where they were joined to a geographic boundary file (also known as a shapefile) for 3139 counties and county equivalents in the United States in 2000. The data were joined using the counties' five-digit Federal Information Processing Standards (FIPS) codes as the primary key. A custom pseudo-projection of the United States on the basis of the Albers equal-area projection was created to depict Alaska and Hawaii in nonstandard geographic locations to the southwest of the United States and facilitate the presentation of the entire 50 states in a concise graphic format.

**Figure 1:**
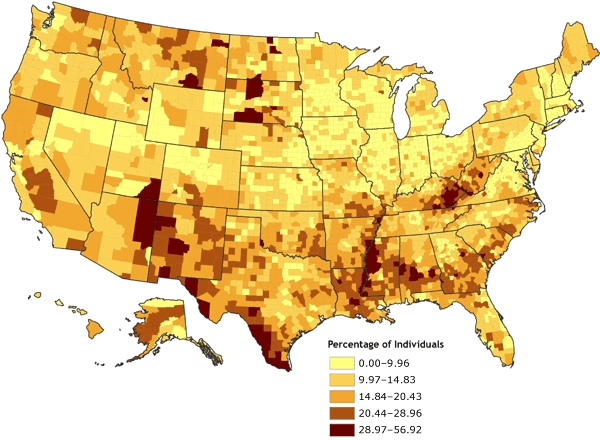
Percentage of individuals living in poverty, by county, 2000. Data are classified by Jenks' optimal (natural breaks) algorithm (11,12). Data source: Community Health Status Indicators (1).

**Figure 2:**
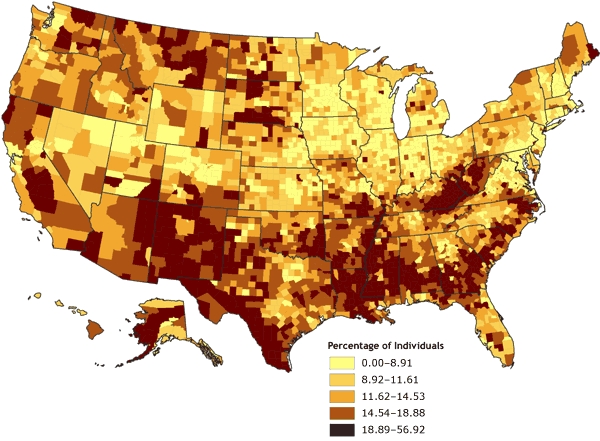
Percentage of individuals living in poverty, by county, 2000. Data are classified by quintiles. Data source: Community Health Status Indicators (1).

**Figure 3:**
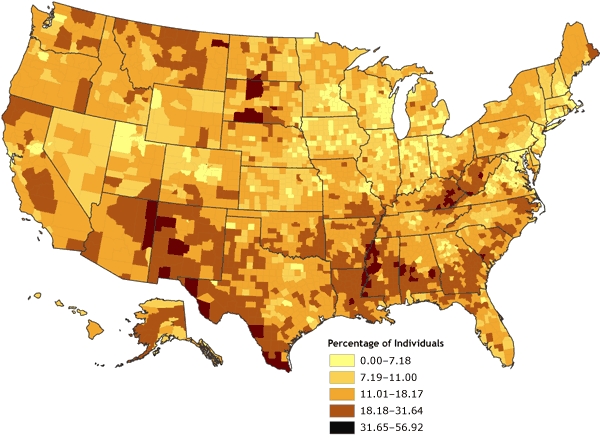
Percentage of individuals living in poverty, by county, 2000. Data are classified by geometric data progression. Data source: Community Health Status Indicators (1).

**Figure 4:**
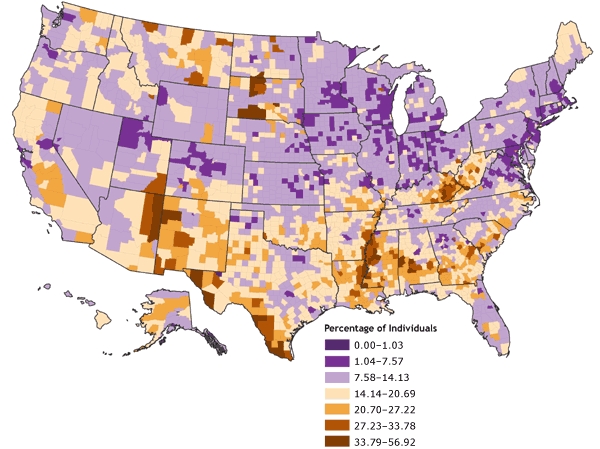
Percentage of individuals living in poverty, by county, 2000. Data are classified by standard deviations from the national mean. Source: Community Health Status Indicators (1).

The county-level rates for poverty were mapped initially using various techniques for determining data cut points. The first map (Figure 1) was derived by classifying the poverty data according to natural breaks, or Jenks' optimal algorithm (11,12), a statistically optimal solution for minimizing within-class variance and maximizing between-class variance. The second map (Figure 2) was derived by using a quintile classification, in which approximately one-fifth of the total number of counties are contained in each of the five data classes. The third map (Figure 3) was derived by using a geometric data classification, in which class breaks are based on class intervals that have a geometrical series. A fourth map (Figure 4), which used a diverging color ramp to emphasize the distribution of the data in reference to the mean of the dataset, was derived through a standard deviation (SD) algorithm. The diverse appearance of Figures 1 through 4 highlights the subjective nature of map construction from the point of view of the cartographer. Although the data were the same for each map, variations in the choices for data cut-point determination and color selection resulted in vastly different appearances. Each map tends to emphasize a different quality or aspect of the data on the basis of the distribution of data values for poverty and the chosen cut points. Without full knowledge and understanding of the underlying choices made by the cartographer, interpretation of these maps can be difficult; the maps can be misleading, either intentionally or by happenstance.

**Table:** 

	**Range, Percentage**				

	**0.00-9.96**	**9.97-14.83**	**14.84-20.43**	**20.44-28.96**	**28.97-56.92**
Number of counties in range	885	1068	723	362	101

**Table:** 

	**Range, Percentage**				

	**0.00-8.91**	**8.92-11.61**	**11.62-14.53**	**14.54-18.88**	**18.89-56.92**
Number of counties in range	630	629	627	628	625

**Table:** 

	**Range, Percentage**				

	**0.00-7.18**	**7.19-11.00**	**11.01-18.17**	**18.18-31.64**	**31.65-56.92**
Number of counties in range	321	803	1300	650	65

**Table:** 

	**Range, Percentage**						

	**0.00-1.03**	**1.04-7.57**	**7.58-14.13**	**14.14-20.69**	**20.70- 27.22**	**27.23-33.78**	**33.79-56.92**
Number of counties in range	1	372	1441	881	312	91	41

On the other hand, as a part of an exploratory data analysis process, the construction of multiple maps, each using different data classification algorithms informed by histograms of the data distributions, can help the analyst gain a better understanding of the data. This understanding, however, is *aspatial* only, and based solely on visual interpretation. The spatial structure in the data cannot be quantified objectively because the classification of data into discrete data ranges involves the analysis of data values in isolation from their spatial context. Although the human brain is capable of recognizing visual patterns, such as those present in a set of mapped data (13), each person's interpretation of the degree and location of such patterns varies.

An alternative and complementary method for exploring the spatial structure of a dataset is to use a statistical measure that accounts for the spatial locations of each data observation in conjunction with the observed data value at each location. One family of such measures was developed to assess and quantify spatial autocorrelation. *Spatial autocorrelation* refers to the degree to which attributes or values at some place on the earth's surface are similar to attributes or values of nearby locations (14). Geographers know this phenomenon as Tobler's first law of geography: "everything is related to everything else, but near things are more related than distant things" (15). If data values that are similar in quantity are also similar in location (e.g., are near one another), the spatial pattern is considered to exhibit positive spatial autocorrelation. Conversely, if data values that are dissimilar are located near one another, the spatial pattern is considered to exhibit negative spatial autocorrelation. Where no correlation exists between data values and their locations, the spatial pattern is considered to exhibit zero spatial autocorrelation.

The two most common measures of spatial autocorrelation are Moran's *I* (16) and Geary's *c* (17). Of the two, Moran's *I* is more commonly used because it is generally considered easier to interpret: its scale is similar to the Pearson correlation coefficient. Following Moran (16) and Waller and Gotway (18), the univariate global Moran's *I* is defined as follows:

I=(1s2)∑i=1N∑j=1Nwij(Yi−Y_)(Yj−Y_)∑i=1N∑j=1Nwij

where

s2=1N∑i=1N(Yi−Y_)2

*Y_i_* and *Y_j_* are data observations at locations *i* and *j*, and *w_ij_* is a spatial weight matrix equal to 1/*d_ij_* in which *d_ij_* represents the Cartesian distances between locations *i* and *j*.

A major limitation of Moran's *I* is that it cannot provide information on the specific locations of spatial patterns; it only indicates the presence of spatial autocorrelation globally. A single overall indication is given of whether spatial autocorrelation exists in the dataset, but no indication is given of whether local variations exist in spatial autocorrelation (e.g., concentrations, outliers) across the spatial extent of the data. To localize the presence and magnitude of spatial autocorrelation, a measure such as Anselin's local indicator of spatial association (LISA) is necessary. LISAs are simply local derivations or disaggregations of global measures of spatial autocorrelation; there are also local versions of Moran's *I* and Geary's *c*. For this study, the local Moran index was used; it is defined for each *i*th location as:

(Yi−Y_)∑j=1Nwij(Yj−Y_)

(18) where *Y_i_* is an observations at each *i*th location, *Y_j_* is an observation at all other locations, and *w_ij_* is a spatial weight matrix equal to 1/*d_ij_* in which *d_ij_* represents the Cartesian distances between the *i*th and *j*th points. A spatial weight matrix can be defined either by contiguity (whether polygons share common boundaries or vertices) or distance (whether polygon geometric centroids are within certain distance thresholds). If distance is used, the spatial weight matrix can be calculated using either a distance banding algorithm, such as inverse distance or inverse distance squared, or a fixed distance band.

**Figure 5:**
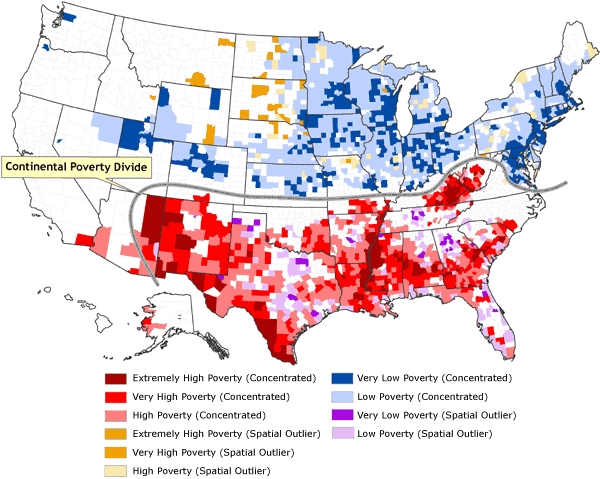
Classification of counties by rate of poverty and spatial situation. The distinctive north–south divide across most of the United States, in which concentrations of low poverty and spatial outliers of high poverty are confined to the northern half, and concentrations of high poverty and spatial outliers of low poverty are confined to the southern half, is termed the *continental poverty divide.* Data source: Community Health Status Indicators (1).

The local Moran's *I* algorithm was used in ArcGIS 9.2 to compute a local Moran value for each county in the United States. Inverse distance weighting with row standardization of the spatial weights, in which each weight is divided by its row sum, was selected; this type of weighting permits comparability among regions with different numbers of neighbors (18). The resulting local Moran indices were converted to *z* scores to indicate whether the similarity or dissimilarity in values between each county and those of its neighbors exceeded the value that would be expected due to chance. Each county was then assigned a categorical value depending on its standardized *z* score, so that each county was one of the following: 1) part of a concentration of counties in which similar levels of poverty clustered; 2) a spatial outlier (i.e., the poverty rate was much different from the poverty rates of nearby or surrounding counties; or 3) neither part of a concentration of counties with similar values or a spatial outlier. These categorical assignments were merged with a categorical assignment of a poverty level based on each county's poverty rate in comparison with the overall mean poverty rate for the United States (Table), and these bivariate categorical values were mapped (Figure 5).

## Results

In 2000, poverty rates for the 3139 U.S. counties and county-equivalents ranged from 0.0% in Loving County, Texas (total population in 2000 = 67), to 56.9% in Buffalo County, South Dakota (total population in 2000 = 2032). The mean county poverty rate was 14.2%, and the median was 13.0%; thus, the distribution was positively skewed. Of the 3139 counties, 2320 had poverty rates within 1 SD of the mean; 376 counties had poverty rates between −1 and −2 SDs; one county (Loving County, Texas) had a poverty rate below −2 SDs; 311 counties had poverty rates between +1 and +2 SDs; 91 counties had rates between +2 and +3 SDs; and 41 counties had poverty rates exceeding +3 SDs (Figure 4).

Spatial clustering of poverty is visually apparent in Figure 4. Dark purple areas indicate counties with low poverty rates (less than 7.57%), corresponding to at least 1 SD below the mean poverty rate. Dark orange areas indicate counties with very high poverty rates (greater than 27.23%), corresponding to greater than 2 SDs above the mean poverty rate. In general, high-poverty clusters occur in the southern United States and in the northern Great Plains states. Low-poverty areas generally appear in the northeastern United States, the Great Lakes states, and the central Rocky Mountain states. Exceptions to these generalizations are apparent from visual inspection of Figure 4.

In Figure 5, the poverty rates are remapped as bivariate categorical values that combine both the poverty rate and the degree of localized spatial autocorrelation in the poverty data. Ten bivariate combinations are depicted: extremely high poverty (concentrated), very high poverty (concentrated), high poverty (concentrated), extremely high poverty (spatial outlier), very high poverty (spatial outlier), high poverty (spatial outlier), very low poverty (concentrated), low poverty (concentrated), very low poverty (spatial outlier), and low poverty (spatial outlier); the remaining counties are neither spatial outliers nor part of a concentrated cluster. The Table provides details of how each county was assigned categorical values for poverty and spatial dimensions and how many counties were included in each bivariate category. Also evident in Figure 5 is a distinctive north–south divide across most of the United States, in which concentrations of low poverty and spatial outliers of high poverty are confined to the northern half, and concentrations of high poverty and spatial outliers of low poverty are confined to the southern half. This divide can be thought of as a *continental poverty divide*, analogous to the more familiar topographic continental divide, which runs orthogonal to this demarcation of poverty and serves as a constraint on the westward extent of the continental poverty divide.

Of the 3139 U.S. counties, 1629 (51.9%) were categorized as belonging to a spatial concentration, whereas only 244 (7.8%) were categorized as being spatial outliers. The remaining 1266 counties (40.3%) were neither. The number of spatially concentrated low-poverty and very low-poverty counties (945) exceeded the number of spatially concentrated high-poverty, very high-poverty, and extremely high-poverty counties (684). Similarly, there were more low-poverty and very low-poverty spatial outliers (161) than there were high-poverty, very high poverty, and extremely high-poverty spatial outliers (83).

**Figure 6:**
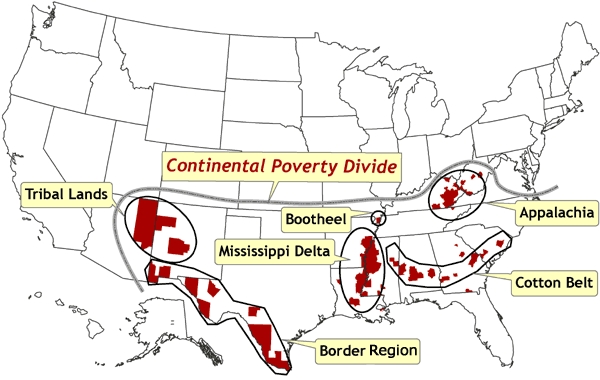
Location of counties that represent spatial clusters in which poverty rates are at least two standard deviations higher than the national mean. These counties correspond with areas that have been defined for other historical, geographic, economic, and cultural reasons (e.g., Appalachia, Mississippi Delta). The *continental poverty divide* is defined as the distinctive north–south divide across most of the United States, in which concentrations of low poverty and spatial outliers of high poverty are confined to the northern half, and concentrations of high poverty and spatial outliers of low poverty are confined to the southern half. Data source: Community Health Status Indicators (1).

The geographic context of these characterizations is readily apparent in Figures 6 through 9.Figure 6 depicts only the spatial concentrations or clusters of counties in which poverty rates are at least 2 SDs higher than the national mean. These areas of very or extremely high poverty generally correspond to areas that have been defined for other historical, geographic, economic, and cultural reasons as Appalachia, the Cotton Belt, the Bootheel of Missouri, the Mississippi Delta, the border region with Mexico, and tribal lands in the Four Corners region.

**Figure 7:**
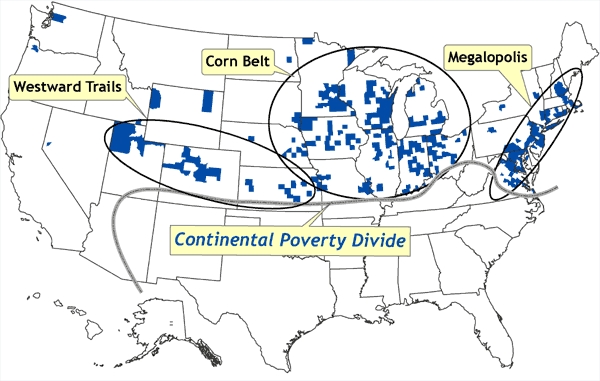
Location of counties that represent spatial clusters in which poverty rates are at least one standard deviation lower than the national mean. These areas of very low poverty correspond to the northeastern megalopolis of urban centers stretching from Richmond, Virginia, to metropolitan Boston; the Corn Belt of the Great Lakes states and the upper Midwest; and a region referred to here as Westward Trails, corresponding to a line of urban centers stretching from Kansas and Nebraska through Colorado to Utah. The *continental poverty divide* is defined as the distinctive north–south divide across most of the United States, in which concentrations of low poverty and spatial outliers of high poverty are confined to the northern half, and concentrations of high poverty and spatial outliers of low poverty are confined to the southern half. Data source: Community Health Status Indicators (1).

Spatial concentrations of counties in which poverty rates are at least 1 SD lower than the national mean are depicted in Figure 7. These areas of very low poverty correspond to the northeastern megalopolis of urban centers stretching from Richmond, Virginia, to metropolitan Boston; the Corn Belt of the Great Lakes states and the upper Midwest; and a region referred to here as Westward Trails, corresponding to a line of urban centers stretching from Kansas and Nebraska through Colorado to Utah.

**Figure 8:**
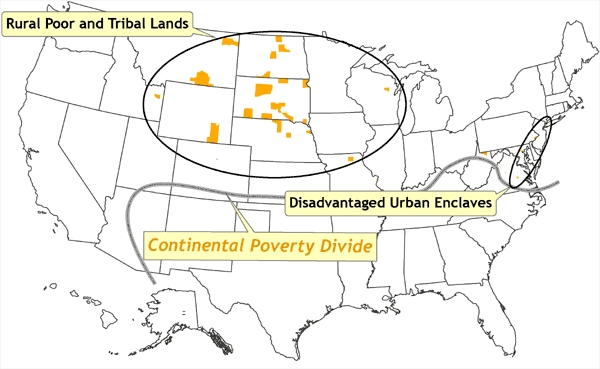
Location of counties in which poverty rates are at least one standard deviation higher than the national mean. These counties are termed *spatial outliers* because they are surrounded by counties in which the poverty rates are well below the national mean. These areas correspond to a few inner cities in the northeast (termed *disadvantaged urban enclaves*) such as Baltimore, Philadelphia, Newark, and New York City, and the rural poor and various tribal lands in the upper Great Plains states. The *continental poverty divide* is defined as the distinctive north–south divide across most of the United States, in which concentrations of low poverty and spatial outliers of high poverty are confined to the northern half, and concentrations of high poverty and spatial outliers of low poverty are confined to the southern half. Data source: Community Health Status Indicators (1).

**Figure 9:**
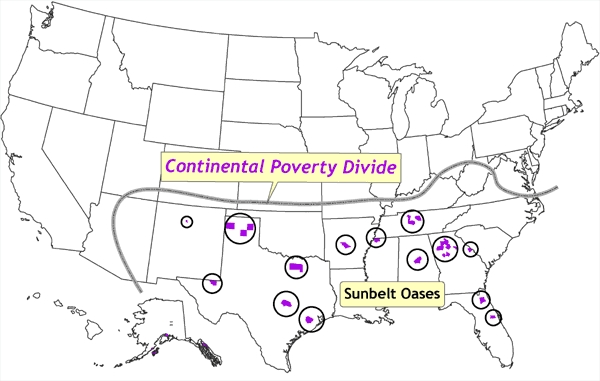
Location of counties in which poverty rates are at least two standard deviations lower than the national mean. These counties are termed *spatial outliers* because they are surrounded by counties in which the poverty rates are well above the national mean. The counties, termed *sunbelt oases,* correspond to rapidly urbanizing areas. The *continental poverty divide* is defined as the distinctive north–south divide across most of the United States, in which concentrations of low poverty and spatial outliers of high poverty are confined to the northern half, and concentrations of high poverty and spatial outliers of low poverty are confined to the southern half. Data source: Community Health Status Indicators (1).

Figure 8 depicts spatial outliers of high poverty, all of which are north of the continental poverty divide. These areas generally correspond to a few inner cities in the northeast (referred to in Figure 8 as *disadvantaged urban enclaves*) such as Baltimore, Philadelphia, Newark, and New York City, and the rural poor and various tribal lands in the northern Great Plains states. Figure 9 depicts spatial outliers of low poverty, all south of the continental poverty divide, corresponding to rapidly urbanizing areas in the south and characterized as *sunbelt oases*.

## Discussion

In its original usage, the word *topography* described the study of place. Over time, usage has evolved to represent the study of landforms. In studying the landscape of poverty in the United States, it is appropriate to resuscitate the original usage of this term while retaining its modern application. Concentrations of high and low poverty are analogous to areas of high and low elevation in the landscape — mountains and broad valleys and deltas. Pockets of high and low poverty (spatial outliers) are analogous to unexpected local variations in elevation — perhaps an isolated hill in an otherwise featureless landscape, a deep depression in a high plateau, or an alpine valley.

Just as with physical landforms, social landscapes result from underlying processes. Mountains are formed because the underlying structure of rocks and minerals are moved into place by vast forces. The spatial extent and local characteristics of mountains are constrained by yet other geophysical forces. Similarly, the landscape of poverty is a result of many forces acting independently and in interaction with other social and structural forces to produce a set of opportunities and constraints. These are manifested in the economic realities of wealth and poverty.

The topography of poverty in the United States is starkly clear. The demarcation between north and south is striking, and isolated pockets of high and low poverty exist within regions that generally have disparate rates of poverty. These observations may be helpful to those who wish to conduct further research into the social and structural forces that result in poverty over geographic regions. The social and structural forces may operate and be observable in these same regions. As with most geographic research, the results of this study are scale dependent. The observations are significant and important at the scale of the United States as a whole, with counties as the units of observation. This limitation, however, is not too restricting as long as researchers postulate that the underlying processes that bring about poverty operate on the same scale. Of course, there are exceptions and local variations, particularly within urban areas. For that reason, it would be appropriate to replicate this analysis at more granular levels of geography, such as at the census tract level within large metropolitan areas.

A second implication of these findings is methodological. Because of the presence of spatially autocorrelated poverty data, care must be exercised in using analytic techniques that rely upon assumptions of the independence of observations, such as ordinary least squares (OLS) regression. These data clearly violate that assumption, and therefore researchers must consider spatial variants to traditional OLS methods, such as spatial regression models and geographically weighted regression (GWR) (19). Another consideration is that the distinctive patterns of localized spatial autocorrelation suggest that there are underlying spatial processes in the study area that may result in spatial nonstationarity of any relationships between the independent and dependent variables in a regression model. GWR techniques have been developed to help deal with this situation.

Because of the recent development of tools and techniques for local spatial analysis, such as LISAs, we can now analyze both spatial patterns of poverty and, perhaps more importantly, the underlying processes involved in forming such spatial patterns. Knowing precisely where concentrations and isolated islands of poverty exist will help social scientists and public health practitioners in their continuing challenge of combating this fundamental threat to health and well-being.

The launch of the CHSI database provides a tremendous resource for public health researchers. It is hoped that this demonstration of exploratory spatial data analysis, using readily available GIS software with spatial statistics capabilities, has highlighted the insights that can be gained from the CHSI dataset. This type of spatial analysis should be considered for data analysis processes for all data with a spatial component and particularly when inferences are to be made from multivariate regression techniques.

## Figures and Tables

**Table.** Assignment of Categorical Values to Dimensions of Poverty and Spatial Concentration in an Analysis Using County-Level Data From the Community Health Status Indicators Project, United States, 2000

Category	Poverty Rate	Local Moran's *z* Score^a^	No. Counties
Extremely high poverty (concentrated)	>3 SDs above mean	≥2.0	110
Very high poverty (concentrated)	Between 2 and 3 SDs above mean	≥2.0	261
High poverty (concentrated)	Between 1 and 2 SDs above mean	≥2.0	313
Extremely high poverty (spatial outlier)	>3 SDs above mean	≤−2.0	13
Very high poverty (spatial outlier)	Between 2 and 3 SDs above mean	≤−2.0	20
High poverty (spatial outlier)	Between 1 and 2 SDs above mean	≤−2.0	50
Very low poverty (concentrated)	>2 SDs below mean	≥2.0	328
Low poverty (concentrated)	Between 2 and 1 SDs below mean	≥2.0	617
Very low poverty (spatial outlier)	>2 SDs below mean	≤−2.0	26
Low poverty (spatial outlier)	Between 2 and 1 SDs below mean	≤−2.0	135
Other^b^	Within 1 SD of mean	−2.0 to 2.0	1266

a Local Moran indices were converted to *z* scores to indicate whether the similarity or dissimilarity in values between each county and those of its neighbors exceeded the value that would be expected due to chance. Each county was assigned a categorical value depending on its standardized *z* score. A *z* score greater than or equal to 2.0 indicates that the county is part of a concentrated cluster; a *z* score less than or equal to −2.0, a spatial outlier; a *z* score between −2.0 and 2.0, neither part of a concentrated cluster nor a spatial outlier (or, "other").

b The "other" category meets either or both of the criteria for poverty rate and *z* score.
